# Autoregulation of JARID2 through PRC2 interaction with its antisense ncRNA

**DOI:** 10.1186/s13104-020-05348-z

**Published:** 2020-10-30

**Authors:** Diaa Al-Raawi, Aditi Kanhere

**Affiliations:** 1 Tumour Biology Research Program, 57357 Children’s Cancer Hospital, Cairo, Egypt; 2 School of Biosciences, University of Birmingham, Birmingham, B15 2TT United Kingdom; 3 Institute of Systems, Molecular and Integrative Biology, University of Liverpool, Liverpool, L69 3GE United Kingdom

**Keywords:** Polycomb repressive complex-2, JARID2, JARID2-AS1, Antisense ncRNA, Autoregulatory loop

## Abstract

**Objective:**

JARID2 is a member of chromatin-modifying Polycomb Repressive Complex-2 or PRC2. It plays a role in recruiting PRC2 to developmental genes and regulating its activity. JARID2 along with PRC2 is indispensable for normal development. However, it remains unclear how *JARID2* expression itself is regulated. Recently a number of non-protein-coding RNAs or ncRNAs are shown to regulate transcription. An antisense ncRNA, JARID2-AS1, is expressed from the first intron of *JARID2* isoform-1 but its role in regulation of *JARID2* expression has not been investigated. The objective of this study was to explore the role of JARID2-AS1 in regulating JARID2 and consequently PRC2.

**Results:**

We found that JARID2-AS1 is localised in the nucleus and shows anti-correlated expression pattern to that of JARID2 isoform-1 mRNA. More interestingly, data mining approach strongly indicates that JARID2-AS1 binds to PRC2. These are important observations that provide insights into transcriptional regulation of JARID2, especially because they indicate that JARID2-AS1 by interacting and probably recruiting PRC2 participates in an auto-regulatory loop that controls levels of JARID2. This holds importance in regulation of developmental and differentiation processes. However, to support this hypothesis, further in-depth studies are needed which can verify JARID2-AS1-PRC2 interactions.

## Introduction

Polycomb group (PcG) proteins are highly conserved transcriptional repressive regulators required for maintenance of cell identity during normal metazoan development [1, 2]. They mediate the epigenetic gene silencing by catalysing repressive histone modifications. PcG proteins form two major complexes, polycomb repressive complex-1 (PRC1) and -2 (PRC2). PRC2 consists of four core proteins, SUZ12, EED, RbAp46/48 and the catalytic subunit EZH2. PRC2 catalyses mono-, di- and trimethylation of histone H3 at lysine 27 (H3K27me3) [3, 4]. In mammalian cells, it is not yet completely clear how PRC2 is recruited to its sites of action. Multiple proteomic and genetics studies have revealed that additional proteins like JARID2, interact with PRC2, forming different subclasses of PRC2 complex such as PRC2.1 and PRC2.2 [1, 5–18]. JARID2 is important for PRC2 function as it contributes to recruitment of PRC2 to chromatin and modulating enzymatic activity through its N-terminal region [1]. The N-terminal region of JARID2 comprises of a nucleosomal binding domain and a RNA binding domain, which together play a role in PRC2 recruitment to genomic DNA [19–21]. JARID2’s post-translational modifications determine its effect on PRC2 activity [22]. JARID2 is also shown to be crucial for directing PRC2 to PRC1 modified nucleosomes through ubiquitin interaction motif (UIM) at its N-terminus [23].

In recent years, more attention has been given to understanding molecular mechanisms of non-protein-coding RNAs (ncRNAs) as essential factors involved in modulating PRC2 function. Antisense ncRNAs are a subclass of ncRNAs that are produced from the DNA strand opposite to the sense strand encoding mRNA. A number of antisense ncRNAs are shown to regulate transcription of sense strand gene. They can overlap with promoters, exons, introns or transcriptional termination/start sites of sense transcripts and their regulatory effect is dependent on their position vis-à-vis their sense strand partner [24–26]. More than 72% of sense transcripts in humans and mice have antisense partners [25, 27, 28]. Antisense RNAs contribute to several biological processes such as RNA editing, stability, alternative splicing, histone modifications and translational regulation [25, 26].

An antisense ncRNA, JARID2-AS1, is expressed in antisense direction to *JARID2* gene located at human chromosome 6. However, its function in regulating JARID2 isoform-1 expression has not been explored. Here, we studied expression of JARID2-AS1 and propose an interesting possibility that JARID2 isoform-1 might auto-regulate its own transcription through the antisense RNA.

## Main text

### Materials and methods

#### Cell culture

HaCaT, a spontaneously immortalized human keratinocyte cell line, HEK293T, a human embryonic kidney cell, A549 cells, a human lung carcinoma epithelial cells, Hela cells, a human cervical cancer cells, HepG2, a Hepatocellular carcinoma and metastatic breast cancer MDA-MB231 were grown in Dulbecco’s modified eagle medium. Human chronic myelogenous leukemia, K562 was grown in RPMI containing 10% foetal bovine serum and 1% penicillin–streptomycin (10.000U/mL) in a 5% humidified CO2 incubator at 37 °C. HaCaT cells were grown as per previously published protocol [29].

HaCaT cell line was kindly donated by Dr. Hotchin, University of Birmingham (UoB) [30]. HEK293T cells were obtained from Dr Tomlinson, UoB [31]. A549 cells and MDA-MB231 cell lines were a gift from Dr. Rappoport (UoB), originally obtained from Public Health England Culture Collections (PHECC), London UK. HepG2 cells were donated by Dr. Michelangeli, UoB and were originally obtained from American Type Culture Collection (ATCC) [32]. K562 cell were originally obtained from ATCC by Dr. Khanim, UoB [33]. All cell lines were regularly mycoplasma tested and authenticated.

#### Nuclear and cytosolic fractionation

To prepare nuclear and cytosolic fractions, 1x10^7^cells were lysed using cold RLN buffer (50 mM Tris–HCl, PH 8.0, 1.5 mM MgCl2, 140 mM NaCl, 0.5% NP-40). After incubating on ice for 10 min, the lysate was centrifuged at 3700 rpm for 5 min at 4 °C. The supernatant was transferred to a new tube and kept as the cytosolic fraction. The pellet (nuclear fraction) was washed with cold RLN buffer. RNA was extracted from both fractions using the RNeasy Mini Kit (Qiagen, Germany). The quality of fractionation was tested by standard agarose gel.

#### RNA extraction, cDNA synthesis and PCRs

Total RNA was extracted from cultured cells, treated with Amplification Grade DNase I (Sigma) to remove genomic DNA and quantified by NanoDrop. Complementary DNA (cDNA) was synthesised using Tetro cDNA synthesis kit (Bioline). For Reverse transcriptase-polymerase chain reaction (RT-PCR), cDNA samples were run using forward and reverse primers (Additional file 1: Table S1) and Red mix reagent (MyTaq Red Mix, Bioline) using a PCR machine (Primus, VWR). Quantitative real-time PCR (qRT-PCR) was performed in triplicate using 100 ng of cDNA, SensiFAST SYBR Hi-ROX kit (Bioline). The values were normalized to 18S rRNA or Actin using ∆∆CT method.

### Results

#### JARID2-AS1 expression is anti-correlated to that of JARID2

**Fig. 1:**
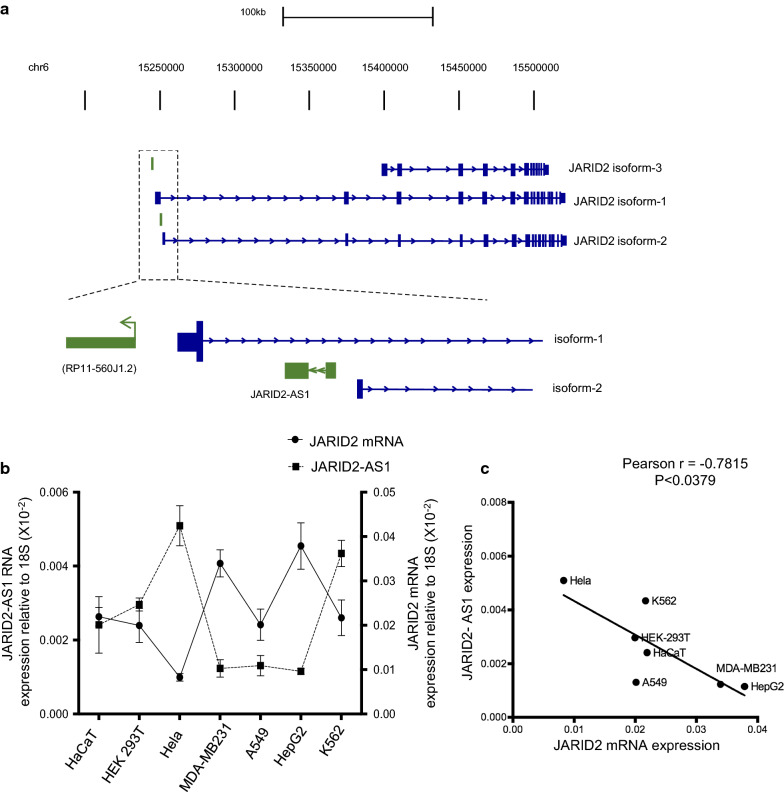
JARID2-AS1 is anti-correlated to JARID2 **a** Pictorial representation of genomic position of JARID2-AS1, showing that it is expressed from the intronic region of JARID2 isoform-1 in the antisense direction. It is also located at the promoter region of JARID2 isoform-2. In the figure, ncRNAs are indicated in green and JARID2 isoforms are shown in blue. Exons are shown as boxes, introns are shown as horizontal lines and arrows describe the direction of transcription. **b** JARID2-AS1 expression as compared to JARID2 isoform-1 mRNA in many different cell types. Expression was measured using qRT-PCR and normalised with respect to 18S rRNA. Data is expressed as mean ± SE. **c**. JARID2 expression is anti-correlated to JARID2-AS1 (r = − 0.7815, *P < 0.05)

Although it is clear that JARID2 plays a vital role in regulating transcription during development and differentiation, we still do not understand how JARID2’s transcription is regulated. We observed that a ncRNA, JARID2-AS1, is expressed in an antisense direction to *JARID2* gene. JARID2-AS1 (Ref-Seq ID: NR_120502) is encoded from the first intron between the transcription start sites of two isoforms of JARID2, isoform-1 and isoform-2 (Fig. 1a). JARID2-AS1 is fully contained within the first intron of JARID2 isoform-1 and is located at the promoter region of the second isoform of JARID2. According to current human gene annotations, JARID2-AS1 has two exons, with a total length of 517 bp.

To analyse the expression of JARID2-AS1 and examine its relation to expression pattern of JARID2 mRNA, RNA samples were extracted from different cell types including; HaCaTs, HEK293T, Hela, MDA-MB231, A549, HepG2 and K562 cells. We have previously shown that JARID2 isoform-1 is the predominant isoform and its other isoforms are expressed at very low levels [29]. Therefore, for this analysis we concentrated only on isoform-1. The expression levels of JARID2 isoform-1 mRNA as well as JARID2-AS1 were measured using qRT-PCR, showing that both JARID2 and JARID2-AS1 were variably expressed in several cell types (Fig. 1b, c). This suggests that these two RNAs are expressed at different levels in different cells and this might relate to their function.

Antisense transcripts can regulate sense transcripts in either a positive or negative manner [24, 25, 34]. Therefore, a co-expression pattern can give a good indication about the regulatory relationship between an antisense RNA with its sense mRNA. Hence, we compared expression levels of JARID2 isoform-1 mRNA with that of JARID2-AS1 in the same cell types. Interestingly, we observed a negative correlation between JARID2-AS1 and JARID2 isoform-1 mRNA in most cell types (Fig. 1 b, c). These results suggest that JARID2-AS1 might have negative effect on JARID2 isoform-1 transcription.

#### JARID2-AS1 and JARID2 isoform-1 expression are negatively correlated during differentiation

**Fig. 2:**
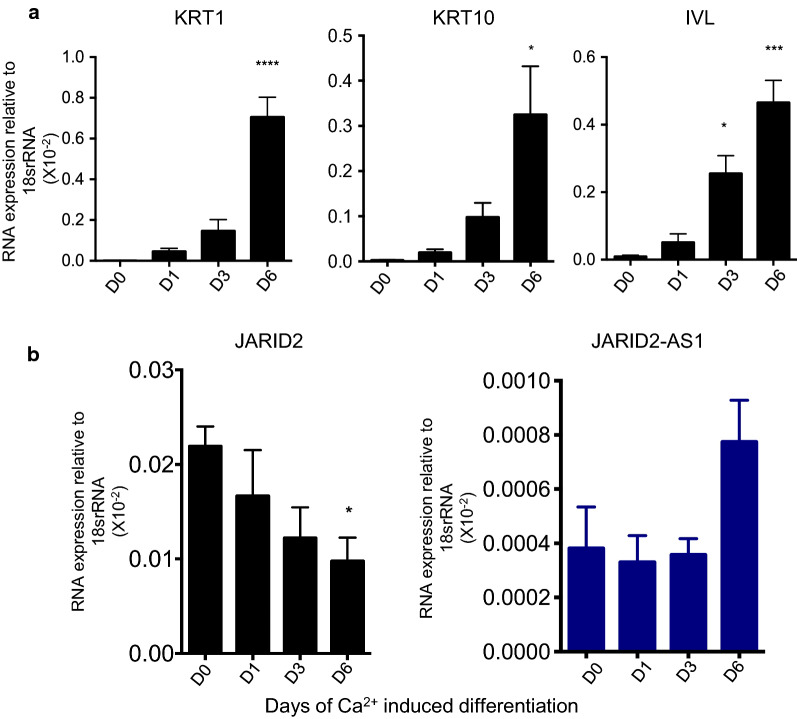
JARID2-AS1 is expressed in differentiated keratinocytes and display an expression pattern opposite to that of JARID2 mRNA. **a** qRT-PCR data showing RNA levels of differentiation markers, KRT1, KRT10 and IVL on days 0, 1, 3, 6 following calcium-induced differentiation of keratinocytes. **b** Expression of JARID2 mRNA as well as JARID2-AS1 was measured using qRT-PCR during keratinocytes differentiation (same as in a). Data is expressed as mean ± SE. Multiple comparison analysis was performed using one-way ANOVA in three independent experiments (n = 3). *P value < 0.05

Given the importance of JARID2 in regulating the cell differentiation, it is important to explore the relationship between JARID2-AS1 and JARID2 isoform-1 in a differentiation model. Our data shows that JARID2-AS1 is expressed in keratinocytes (Fig. 1b, c). In addition, JARID2 plays an important role in regulating epidermal cell differentiation [29, 35]. To test this, we utilized an in vitro keratinocyte differentiation model, where HaCaT cells were grown in a high Ca^2+^ medium for 6 days. We confirmed successful differentiation using keratinocyte differentiation markers, Keratin-1(KRT1), Keratin-10 (KRT10) and Involucrin (IVL) (Fig. 2a). Our results demonstrate that expression of JARID2-AS1 is significantly upregulated in differentiated keratinocytes when compared to undifferentiated keratinocytes. This is in contrast to JARID2 mRNA which is at lowest level upon 6 days of differentiation (Fig. 2b). This data again shows that JARID2 mRNA levels are anti-correlated to JARID2-AS1 during differentiation, suggesting that JARID2-AS1 might negatively regulate the transcription of JARID2 isoform-1.

#### JARID2-AS1 is a nuclear-enriched long ncRNA

**Fig. 3:**
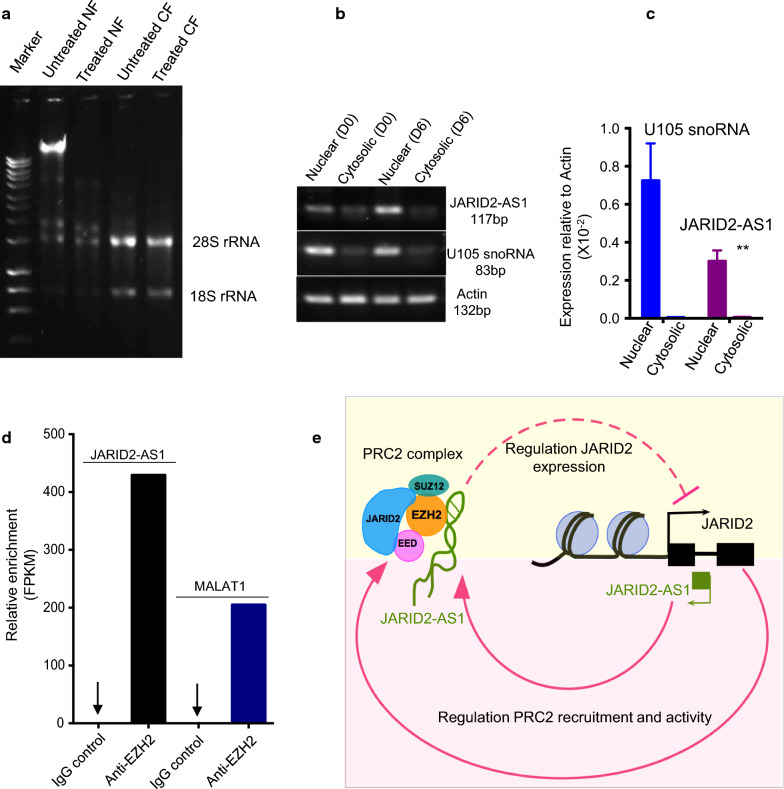
JARID2-AS1 is enriched in the nucleus, it interacts with PRC2 and might be involved in auto-regulation of JARID2 **a** Agarose gel showing untreated and treated RNA with DNase I to remove genomic DNA from nuclear (NF) and cytosolic (CF) fractions. Ribosomal RNA bands are seen only in CF. **b** JARID2-AS1 is enriched in the nucleus in both undifferentiated (D0) and differentiated (D6) HaCaTs. **c** qRT-PCR data also shows JARID2-AS1 is significantly enriched in the nucleus. U105 snoRNA was used as nuclear control, whereas actin was used as equal loading control. Relative expression (n = 3) was normalized to actin and analysed using the student’s *t* test (** P value < 0.01). **d** RNA–protein binding data (accession number GSE36070) was analysed and showed that JARID2-AS1 is enriched in EZH2 bound RNAs. MALAT1 was previously shown to interact with EZH2 in many cancers, therefore, it is used as positive control. **e** A model showing autoregulation of JARID2. JARID2 protein is a known regulatory partner of PRC2 complex. We hypothesize that JARID2-AS1 might negatively regulate the transcription of JARID2 isoform-1 by interacting with EZH2 and JARID2 thus forming an auto-regulatory loop

While mRNAs are synthesized in the nucleus and transported to the cytoplasm where they are translated into proteins, most long ncRNAs (lncRNAs) are localized in the nucleus, in accordance with their roles in regulation of transcription [36, 37]. To examine the cellular localization of JARID2-AS1, RNA samples were extracted from the nucleus and cytoplasm of undifferentiated keratinocytes at day 0 (D0) and differentiated keratinocytes at day 6 (D6). The quality of nuclear and cytosolic RNA fractionation was tested using a gel electrophoresis (Fig. 3a). U105, a small nucleolar RNA (snoRNA), was used as a nuclear marker, while actin was used as a loading control as it is equally present in both nuclear and cytosolic fractions. Agarose gel electrophoresis showed that JARID2-AS1 is highly enriched in the nuclear fraction (NF) of both undifferentiated (D0) and differentiated (D6) keratinocytes (Fig. 3b, Additional file 2: Figure S1). To quantify this finding, the localization of JARID2-AS1 was further investigated using qRT-PCR. This experiment confirmed that JARID2-AS1 was significantly enriched in the nucleus (Fig. 3c). This may reflect its role in regulating nuclear processes such as transcription.

#### JARID2-AS1 interacts with polycomb protein EZH2

Based on the anti-correlation in the expression pattern between JARID2-AS1 and JARID2 isoform-1 mRNA as well as the nuclear localization of JARID2-AS1, we hypothesize that JARID2-AS1 might be involved in regulation of JARID2 transcription. A number of antisense RNAs are shown to regulate the sense transcription by binding to PRC2 protein, EZH2 [38–42]. To explore the mechanism by which this ncRNA might regulate the expression of JARID2 isoform-1, we mined EZH2-RNA interaction data (accession number GSE36070 [43]). This data was generated using iCLIP (Cross-Linking Immunoprecipitation and RNA-sequencing) method where in vivo RNA–protein interactions are cross-linked and pulled-down using EZH2 antibody, followed by RNA purification and processing for sequencing [44, 45].

In this data, JARID2-AS1 is highly enriched in EZH2 pull-down compared to IgG control (Fig. 3d). Multiple studies have shown that MALAT1, a highly expressed lncRNA in many cancer types, interacts with EZH2 [46–48]. Therefore, MALAT1 served as positive control for this analysis (Fig. 3d). However, further scrutiny would be required to confirm this interaction.

### Discussion

JARID2 is an important transcriptional regulator that plays an essential role during early development. Without JARID2, embryonic stem cells cannot differentiate. Although it is very clear that JARID2 plays a crucial role in regulating transcription of differentiation genes [13–15, 17, 22, 29, 35], it is still not clear which factors and mechanisms that regulate JARID2 levels during differentiation.

Antisense ncRNAs play a key role in regulating expression levels of coding genes through epigenetic silencing, transcription and mRNA stability via pairing with protein coding mRNAs, thus forming sense-antisense hybrids [25, 27, 49]. JARID2-AS1, a ncRNA, is expressed in antisense direction from *JARID2* locus (Fig. 1a). However, its relation to JARID2 expression has not been studied. Through data-mining approach, we found that JARID2-AS1 interacts with EZH2 (Fig. 3d). We also found that JARID2-AS1 expression is anti-correlated with the expression of JARID2 mRNA (Figs. 1, 2). In addition, these findings suggest that JARID2-AS1 might downregulate the expression of JARID2 isoform-1 during cell differentiation (Fig. 2). One potential mechanism is that JARID2-AS1 may interfere or inhibit JARID2 transcription. In this scenario, we will expect that this antisense transcript to be localized in the nucleus as we observe in the case of JARID2-AS1(Fig. 3a–c). Several repressive ncRNAs such as XIST and HOTAIR are shown to interact and recruit PRC2 proteins such as SUZ12 or EZH2 [50]. Like XIST and HOTAIR, JARID2-AS1 might regulate JARID2 expression by recruiting EZH2 at *JARID2* gene promoter. A similar mechanism is observed in case of transcription regulation of lymphoid enhancer binding factor, LEF1, gene that is expressed during epithelial-to-mesenchymal transition. Like JARID2-AS1, antisense LEF1 ncRNA overlaps with the first intron of LEF1 transcript and is enriched in the nucleus. It has been reported that antisense LEF1 transcript regulates the expression of LEF1 in *Cis* through binding to the promoter of LEF1 and PRC2. This allows binding of PRC2 to the LEF1 promoter, leading to the blocking of LEF1 transcription [41]. HOXD-AS1, a nuclear antisense RNA, is another example of antisense RNAs that control gene transcription through PRC2. It inhibits transcription of HOXD3 through depositing H3K27me3 on its promoter [51].

Studies have reported that a tripartite interaction between PRC2, RNA and other proteins like JARID2 are needed for proper localization of PRC2 on target genes [21] as well as for enhancing the catalytic activity of EZH2 [52]. In this tripartite interaction, JARID2 acts as a surface for binding of RNA to PRC2 [53]. A further study shows that RNA inhibits the enzymatic activity of both PRC2 subclasses through its binding to regulatory sites on the PRC2 complex [54]. Together these observations suggest an auto-regulatory loop (Fig. 3e). This hypothesis provides an explanation for rapid down-regulation of levels of developmentally important gene such as JARID2.

## Limitations

The strength of current study is that it examines a novel ncRNA overlapping PRC2 component JARID2 and presents an autoregulatory model which might be applicable to other PRC2 genes in addition to JARID2. However, a further careful scrutiny of this hypothesis is needed. It would be necessary to knockout JARID2-AS1 and study its effects on JARID2 expression. The interaction of JARID2-AS1 with EZH2 as well as with JARID2 needs further verification using RNA-pulldown approaches.

## Supplementary information

**Additional file 1: Table S1.** Primers used for qRT-PCR.

**Additional file 2: Figure S1.** Full gel pictures corresponding to main Fig. 3b. JARID2-AS1 is enriched in the nucleus in both undifferentiated (D0) and differentiated (D6) HaCaTs. U105 snoRNA was used as nuclear control, whereas actin was used as equal loading control.

## Data Availability

The data used and analysed during the present study are available from AK on reasonable request.

## Abbreviations

PcG: Polycomb group proteins
PRC1: Polycomb repressive complex-1
PRC2: Polycomb repressive complex-2
H3K27me3: Histone H3 at lysine 27
UIM: Ubiquitin Interaction Motif
ncRNAs: Non-protein-coding RNAs or non-coding RNAs
cDNA: Complementary DNA
qRT-PCR: Quantitative real-time PCR
RT-PCR: Reverse transcriptase-polymerase chain reaction
KRT1: Keratin-1
KRT10: Keratin-10
IVL: Involucrin
mRNAs: Messenger RNAs
D0: Day 0
D1: Day 1
D3: Day 3
D6: Day 6
NF: Nuclear Fraction
CF: Cytosolic Fraction
