# Supplementary material for: Autoregulation of JARID2 through PRC2 interaction with its antisense ncRNA
Source: BMC Res Notes. 2020 Oct 30;13:501. doi: 10.1186/s13104-020-05348-z (PMC7602346; doi:10.1186/s13104-020-05348-z)
Supplement: Supplementary file 1 — Additional file 1: Table S1. Primers used for qRT-PCR. [file 13104_2020_5348_MOESM1_ESM.pdf]

Supplementary Table 1. Primers used for qRT-PCR.

**Gene**	**Accession number**	**Sequence of forward (F) and reverse (R) primers (5'-3')**	**Product size (bp)**
JARID2	NM_004973.3	F: GTCCCCTTTTGCAATCAGCA<br>R:TCCCATCACTGTCATCGTATTTCT	94
JARID2-AS1	NR_120502.1	F:CTGCATCCGAAACGGACGTG<br>R: CAGAGGGCTGGGTAGAATGC	117
IVL	NM_005547.2	F: TGTGAGTCTGGTTGACAGTAGC<br>R: GGAGGAACAGTCTTGAGGAGC	103
KRT1	NM_006121.3	F: GTTCCAGCGTGAAGTTTGTT<br>R: TAAGGCTGGGACAAATCGAC	142
KRT10	NM_000421.3	F: GCAAATTGAGAGCCTGACTG<br>R: CAGTGGACACA TTTCGAAGG	86
U105 snoRNA	NR_004381	F:CCCTATCTCTCATGATGAACACAT<br>R: CCCATCTCTTCTTCAGAGCG	83
18S rRNA	NR_145820.1	F: GCAATTATTCCCCATGAACG<br>R: GGCCTCACTAAACCATCCAA	123
ACTB	NM_001101.3	F: ATGATGATATCGCCGCGCTC<br>R: CCACCATCACGCCCTGG	132
