# Supplementary material for: Autoregulation of JARID2 through PRC2 interaction with its antisense ncRNA
Source: BMC Res Notes. 2020 Oct 30;13:501. doi: 10.1186/s13104-020-05348-z (PMC7602346; doi:10.1186/s13104-020-05348-z)
Supplement: Supplementary file 2 — Additional file 2: Figure S1. Full gel pictures corresponding to main Fig. 3b. JARID2-AS1 is enriched in the nucleus in both undifferentiated (D0) and differentiated (D6) HaCaTs. U105 snoRNA was used as nuclear control, whereas actin was used as equal loading control. [file 13104_2020_5348_MOESM2_ESM.pptx]

## Slide 1
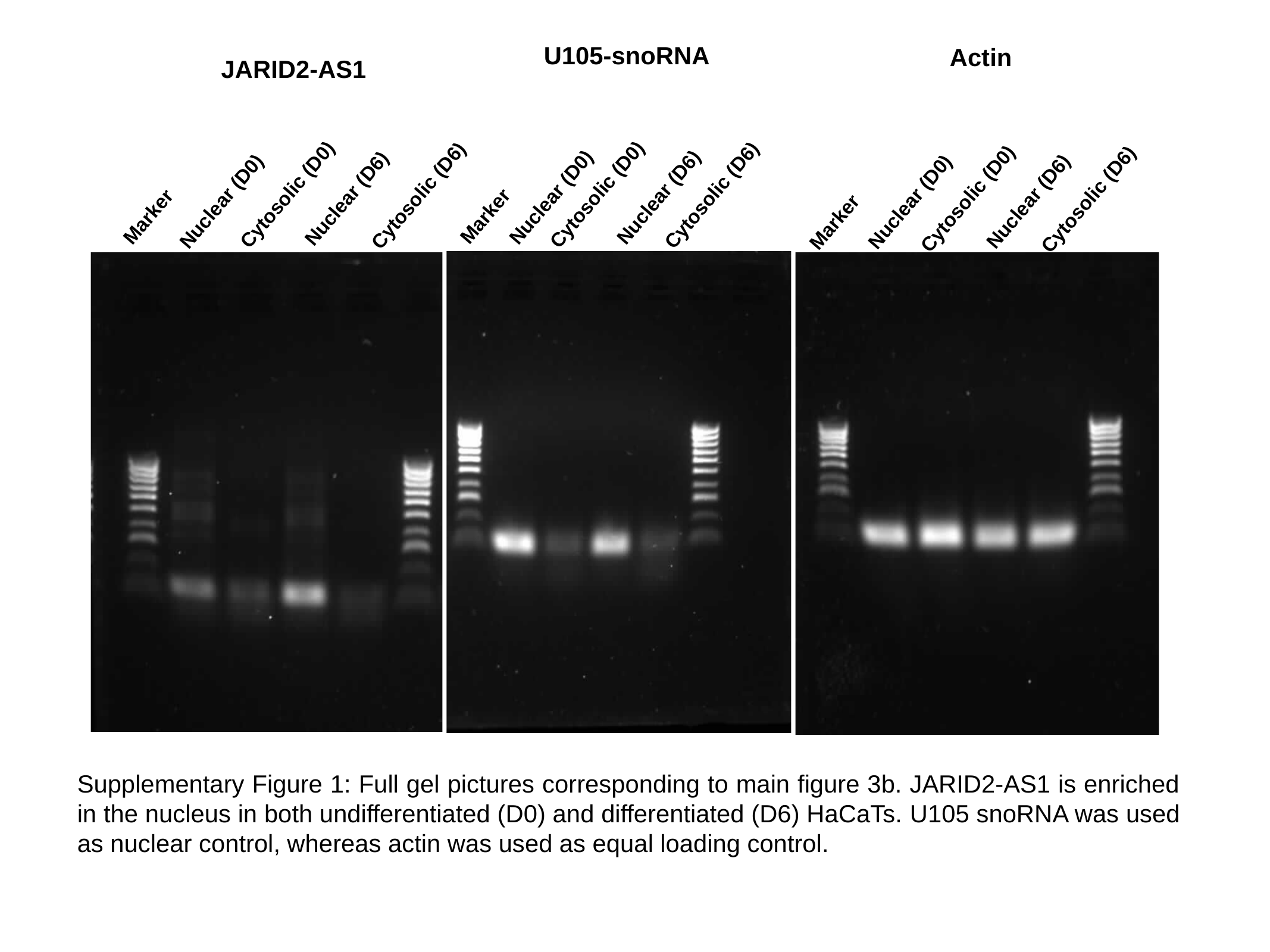

U105-snoRNA
Actin
JARID2-AS1
Nuclear (D6)
Nuclear (D0)
Nuclear (D6)
Cytosolic (D0)
Cytosolic (D0)
Cytosolic (D6)
Nuclear (D6)
Nuclear (D0)
Cytosolic (D6)
Nuclear (D0)
Cytosolic (D0)
Cytosolic (D6)
Marker
Marker
Marker
Supplementary Figure 1: Full gel pictures corresponding to main figure 3b. JARID2-AS1 is enriched in the nucleus in both undifferentiated (D0) and differentiated (D6) HaCaTs. U105 snoRNA was used as nuclear control, whereas actin was used as equal loading control.
